# Supplementary material for: Identification of photoperiod-induced specific miRNAs in the adrenal glands of Sunite sheep (Ovis aries)
Source: Front Vet Sci. 2022 Jul 22;9:888207. doi: 10.3389/fvets.2022.888207 (PMC9354845; doi:10.3389/fvets.2022.888207)
Supplement: Supplementary file 1 [file Data_Sheet_1.ZIP › Supplementary file/miRDeep2 file.zip!/miRDeep2 file/Novel_Structure/SP42A1/Novel_67.pdf]

Provisional ID	:	NC_019459.2_4326
Score total	:	1.2
Score for star read(s)	:	-1.3
Score for read counts	:	0
Score for mfe	:	0.9
Score for randfold	:	1.6
Score for cons. seed	:	
Total read count	:	17
Mature read count	:	17
Loop read count	:	0
Star read count	:	0

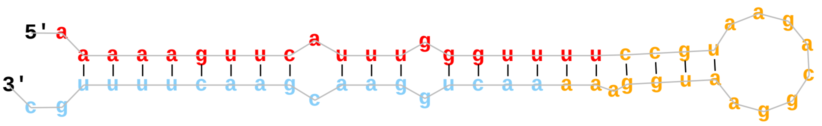

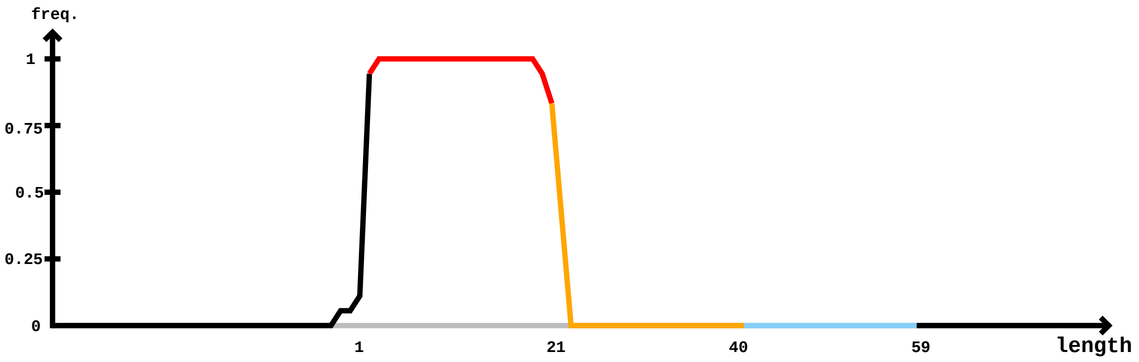

Mature Star

```
5'- ccucuggcucuaucauugucgugaguuggucaaaaaguucauuuggguuuuccguaagacggaauggaaaaacuggaacgaacuuuugcccaauccaguacacagccu   -3'    exp
    .....((((.......(((..((.(((((.(((((.((((.(((.((((((((((........)))).)))))).))).))))))))).))))).))..))).)))).    reads    mm    sample
    ............................gucaaaaaAuucauuuggguu...........................................................    1        1     seq
    ..............................caaaaaguucGuuuggguuuuc........................................................    1        1     seq
    ...............................aaaaaguucGuuuggguuu..........................................................    2        1     seq
    ...............................aaaaaguucGuuuggguuuu.........................................................    12       1     seq
    ...............................aaaaGguucauuuggguuuu.........................................................    1        1     seq
    ................................aaaGguucauuuggguuuu.........................................................    1        1     seq
```
